# A Rose by Any Other Verb: The Effect of Expectations and Word Category on Processing Effort in Situated Sentence Comprehension

**DOI:** 10.3389/fpsyg.2021.661898

**Published:** 2021-05-28

**Authors:** Les Sikos, Katharina Stein, Maria Staudte

**Affiliations:** Language Science and Technology, Saarland University, Saarbrücken, Germany

**Keywords:** processing effort, expectations, situated surprisal, visual world paradigm, language comprehension, referential uncertainty, grammaticality maze, pupillometry

## Abstract

Recent work has shown that linguistic and visual contexts jointly modulate linguistic expectancy and, thus, the processing effort for a (more or less) expected critical word. According to these findings, uncertainty about the upcoming referent in a visually-situated sentence can be reduced by exploiting the selectional restrictions of a preceding word (e.g., a verb or an adjective), which then reduces processing effort on the critical word (e.g., a referential noun). Interestingly, however, no such modulation was observed in these studies on the expectation-generating word itself. The goal of the current study is to investigate whether the reduction of uncertainty (i.e., the generation of expectations) simply does not modulate processing effort-or whether the particular subject-verb-object (SVO) sentence structure used in these studies (which emphasizes the referential nature of the noun as direct pointer to visually co-present objects) accounts for the observed pattern. To test these questions, the current design reverses the functional roles of nouns and verbs by using sentence constructions in which the noun reduces uncertainty about upcoming verbs, and the verb provides the disambiguating and reference-resolving piece of information. Experiment 1 (a Visual World Paradigm study) and Experiment 2 (a Grammaticality Maze study) both replicate the effect found in previous work (i.e., the effect of visually-situated context on the word which uniquely identifies the referent), albeit on the verb in the current study. Results on the noun, where uncertainty is reduced and expectations are generated in the current design, were mixed and were most likely influenced by design decisions specific to each experiment. These results show that processing of the reference-resolving word—whether it be a noun or a verb—reliably benefits from the prior linguistic and visual information that lead to the generation of concrete expectations.

## 1. Introduction

Recent language processing literature converges on establishing a predictive mechanism in which expectations about upcoming words can be determined by both linguistic and visual contexts. On the one hand, word expectancy, as derived from the linguistic context, has been shown to reliably correlate with processing effort, i.e., more predictable words are easier to process (e.g., Kutas and Hillyard, 1980; Federmeier et al., 2007; Van Berkum et al., 2007; Demberg and Keller, 2008; Smith and Levy, 2013). On the other hand, more recent work has shown that the visual context can also influence linguistic expectancy and, for instance, reduce the processing effort for a word when the co-present scene enables very clear and concrete predictions for that word (Ankener et al., 2018; Tourtouri et al., 2019; Staudte et al., 2021).

For example, Ankener and colleagues examined two critical regions in German stimulus sentences in order to investigate the chain of processing from generating an expectation, to the downstream effects of that expectation. A sentence such as *Die Frau verschüttet jetzt das Wasser* (“The woman spills now the water”) was accompanied by a visual scene depicting four objects, some of which were “spillable.” The two regions of interest within the sentence were: (a) the verb (e.g., *verschüttet*, “spills”), where linguistic expectations for upcoming spillable object nouns were generated, and (b) the sentence-final noun (e.g., *Wasser*, “water”) whose expectancy varied depending on whether one, three, four, or none of the depicted objects could be spilled. To analyze eye movements, new inspections of the target object were extracted during the verb region (i.e., before the target was mentioned). Eye movements indicated that participants were more likely to shift their attention to the target when it was the only spillable object in the display, than when there were three or four spillable objects. Although these results did not distinguish anticipation strength between three and four potential target objects, they do provide evidence for listeners' strong(est) anticipation of the target when it was the only object that matched the verb's selectional restrictions. This suggests that uncertainty about the upcoming referent was reduced by exploiting linguistic knowledge about the verbal restrictions. Results further showed that processing effort at the object noun, as measured by the pupillometric Index of Cognitive Activity (ICA, Marshall, 2002; Ankener et al., 2018) and electrophysiological measures (Staudte et al., 2021), was higher when more spillable objects were co-present. In contrast, the object noun was easiest to process when no other spillable competitors were co-present, and thus the object noun was most predictable. These results demonstrate that processing effort is directly influenced by both visual and linguistic contexts, which together modulate *visually-situated* expectations.

Other work further suggests that words which reduce uncertainty about upcoming linguistic continuations require greater processing effort than words that do not reduce uncertainty (Frank, 2013; Hale, 2016; Linzen and Jaeger, 2016; Maess et al., 2016). Linzen and Jaeger (2016) revealed, for instance, that a word which reduces the uncertainty about possible continuations elicits longer reading times. Maess et al. (2016) measured magnetoencephalography (MEG) while participants listened to simple German sentences in which the verbs either constrained expectations for a particular noun or not (e.g., *Er dirigiert/leitet das Orchester*, “He conducts/leads the orchestra”) and found that more constraining verbs (e.g., *dirigiert*, “conducts”) elicited greater processing difficulty, as reflected in larger N400 amplitudes, than unconstraining verbs (e.g., *leitet*, “leads”). Moreover, when a noun (e.g., “orchestra”) followed a constraining verb, the noun elicited a reduced N400 relative to the same noun following an unconstraining verb, indicating that it was easier to process. This pattern of effects was interpreted as “trade-off” in processing effort between the moment at which a prediction is made and a later point in time when the prediction is cashed out. Although Maess et al. (2016) attribute this difference in processing cost to the constraining word preactivating semantic features of the upcoming predictable noun, the effect is also consistent with the reduction of uncertainty. Lastly, similar trade-off effects, but in the P600 component, were found by Ness and Meltzer-Asscher (2018) and attributed to pre-updating, a mechanism thought to reflect an early integration of the predicted upcoming verb argument.

Interestingly, however, neither measure of processing difficulty in Ankener et al. (2018) or Staudte et al. (2021) indicated a modulation of processing effort at the verb itself, despite the fact that the verb reduced uncertainty about upcoming referents to a greater or lesser extent depending on the visual context. This is somewhat surprising given the results of previous work indicating that more constraining words/verbs elicit greater processing effort than unconstraining words/verbs (Frank, 2013; Hale, 2016; Linzen and Jaeger, 2016; Maess et al., 2016; Ness and Meltzer-Asscher, 2018). In contrast, Ankener and colleagues interpreted their findings as an indication that processing effort at the predictive stage (i.e., at the verb) was simply not affected by the amount of (referential) uncertainty that can be reduced at the verb and that this might be specific to the *situated* and *referential* nature of expectations.

An alternative explanation for the findings of Ankener and colleagues is that the particular word categories used in their stimuli contributed to the pattern of null effects found at the verb and significant effects found at the subsequent noun. More specifically, the linear order of words in Ankener et al. (2018) and Staudte et al. (2021)—in which participants first encountered the verb and then the noun—may have emphasized the referential aspects of the object noun. That is, while nouns in general can be thought of as direct pointers to objects in the world, this function receives particular emphasis when the noun is used to uniquely disambiguate a reference and, consequently, to decode the entire sentence proposition. The verb, in contrast, does not index the displayed objects as directly, and therefore may not strictly exclude objects that do not fit the verb's selectional restrictions. This difference could potentially explain the lack of effects found at the verb.

Thus, the goal of the current study is to disentangle two potential explanations for these previous findings: (1) Is it the case that the generation of expectations—and the resulting reduction of referential uncertainty—simply does not modulate processing effort, as suggested by Ankener and colleagues? Or (2) can the lack of effects found at the verb in these previous studies be better explained by differences in the referential function of nouns and verbs and their linear order of occurrence? We address these questions in two visually-situated experiments that each employ a common German construction in passive voice wherein the mention of the object noun is followed by a past participle form of the verb. This construction allowed us to reverse the linear order of the object noun and the verb, such that the noun now serves to reduce (some) uncertainty while the subsequent participle provides the necessary information for uniquely identifying the target scene/object in the display.

Experiment 1 is similar to Ankener et al. (2018) in that it employs a visual world paradigm design and assesses pupillary measures of processing difficulty: auditory sentences are presented while listeners view scenes depicting actions being performed on objects. Experiment 2 uses a more exploratory design in which participants preview the same scenes as in Experiment 1, but processing difficulty is assessed via word-by-word reading times in the Grammaticality Maze (G-Maze) task (Forster et al., 2009).

Crucially, both experiments find a similarly graded effect of visually-situated context on the word which uniquely identifies the referent (i.e., the verb in the current design). These findings replicate the effect found at sentence-final nouns in Ankener et al. (2018) and Staudte et al. (2021). The results of Experiment 1 are also consistent with Ankener and colleagues in that we find no modulation of processing effort (as indexed by ICA) on the word where expectations are first generated (i.e., the noun in the current design). In contrast, Experiment 2 also reveals a significant effect at the noun. This combined pattern of effects in Experiment 2 is consistent with Maess et al. (2016) and may, among other things, reflect a trade-off in processing effort between expectation generation and reference resolution.

## 2. Experiment 1: Pupillometric Measure of Noun and Verb Processing

The primary goal of Experiment 1 was to investigate whether the expectedness of a verb, as modulated by a co-present visual referential context, predicts processing effort at that verb. In addition, we also examined whether processing effort at the prediction-generating object noun was modulated by the degree to which the noun reduced uncertainty about the upcoming verb. Following Ankener et al. (2018), we assessed processing effort using ICA, a pupillometric measure of cognitive load which is robust to eye movements and changes in ambient lighting (Marshall, 2000, 2002).

### 2.1. Methods

#### 2.1.1. Participants

Thirty-two native speakers of German (22 female; 19–40 years old, *M* = 25.3, *SD* = 4.9) were recruited from Saarland University community and were compensated 7.50€ for their participation. All participants reported normal or corrected-to-normal vision. Due to a technical error, the data from one participant could not be used for analyses.

#### 2.1.2. Materials

Participants listened and responded to pre-recorded sentences (in German) while viewing visual displays. Forty experimental sentences were constructed using the following template: *Sag mir, ob* [artikel objekt], *die von der Figur* [ge-verb-n] *wird*, [position] *ist* (“Tell me if [article object] that by the figure is being [verb-ed] is [position]”). For instance, *Sag mir, ob die Rose, die von der Figur gegossen wird, oben ist* (“Tell me if the rose that the figure is watering is on the top”). Queried positions rotated through five possibilities: *oben/unten/links/rechts/fehlt* (“on the top/bottom/left/right/ missing”). Auditory stimuli were recorded with a natural speaking rate and intonation with Audacity 2.2.14 and annotated with Praat 6.0.37 (Boersma, 2001).

**Figure 1:**
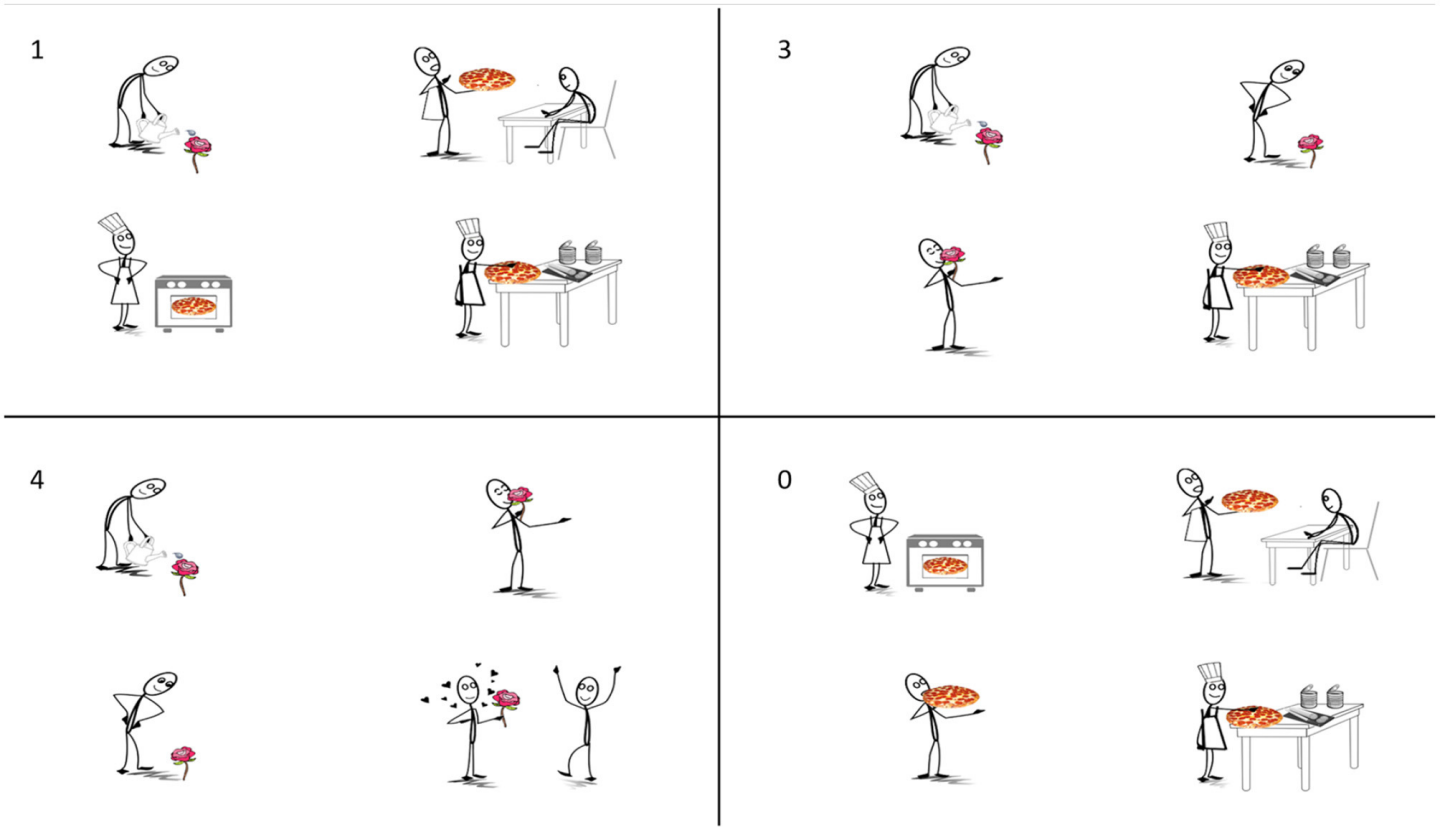
Experiment 1: Example visual display in all four conditions. From **left** to **right** and **top** to **bottom**: *1-match, 3-match, 4-match*, and *0-match* conditions, given the sentence: “Tell me if the rose that the figure is watering is on the bottom”.

The expectedness of the target verb was manipulated by pairing each auditory stimulus with four visual displays in a 1 x 4 design (Figure 1). Each display consisted of four scenes, wherein each scene depicted a different action being performed on an object. Displays differed in the number of scenes (1, 3, 4, or 0) that contained the mentioned object (e.g., *die Rose*; “the rose”)^1^. In the *1-match* condition, the mentioned object was depicted in only one of the four scenes. Thus, upon hearing the object, the target verb (e.g., *gegossen*; “watering”) becomes highly expected. A distractor object (e.g., pizza) was depicted in the remaining three scenes. In the *3-match* condition, the mentioned object was depicted in three of the four scenes, but only one of these scenes was consistent with the target verb. This manipulation decreases the expectedness of the target verb relative to the 1-match condition because upon hearing the object three action verbs were still equally likely. The other two scenes containing the mentioned object served as competitors. The distractor object appeared in the remaining scene. In the *4-match* condition, the mentioned object was depicted in all four scenes, further decreasing the expectedness of the target verb because upon hearing the object four verbs are still possible. Again, however, only one scene was consistent with the target verb. Finally, in the *0-match* condition, the mentioned object did not appear in any of the scenes. Thus, at the point when the object is mentioned, it becomes clear that the visual display cannot provide any information about the target verb. Visual displays were counterbalanced across items such that the mentioned object from one item served as the distractor for another item. For instance, the displays in Figure 1 were also paired with the sentence, *Sag mir, ob die Pizza, die von der Figur belegt wird, oben ist*. (“Tell me if the pizza that the figure is making is on the top.”). Scenes were composed in Paint S (version 5.6.9 (312)5) by arranging images from open source clipart websites (https://openclipart.org; http://clipart-library.com). The position of targets, competitors, and distractors were rotated across items.

In order to disguise the critical manipulation, 40 filler sentences were constructed using three different question structures: one resembled experimental items, one used a verb-subject-object (VSO) construction (e.g., *Verstaut die Figur die Bluse auf der linken Seite?*; “Does the figure package the blouse on the left?”), and one used a relative clause construction (e.g., *Ist der Kugelschreiber, der von der Figur benutzt wird, links?*; “Is the pen that the figure is using on the left?”). Each filler sentence was paired with one visual display consisting of four scenes. Displays differed across filler items in the number of scenes that contained the mentioned object (7 filler displays contained the object in one scene, 12 contained the object in two scenes^2^, 7 contained the object in three scenes, 7 contained the object in four scenes, and 7 contained the object in zero scenes).

Four stimulus lists were created from the above materials according to a Latin square design. Experimental items were counterbalanced across lists such that each participant observed ten items in each condition but no participant observed any item in more than one condition. All participants saw the same fillers. Presentation order was pseudorandomly mixed such that no more than two items of the same condition occurred in sequence. No objects or verbs were repeated across experimental or filler items.

#### 2.1.3. Procedure

Participants were randomly assigned to a stimulus list (8 per list). Following informed consent participants were seated approximately 60 cm from a computer monitor and an Eye-Link 1000+ (SR Research, Ltd.; Mississauga, Ont., Canada). Participants completed a brief, self-paced familiarization session that introduced all the actions that would later appear in the visual displays, but with different objects than in the experimental trials. Each action appeared one at a time while an auditory recording of the corresponding verb was played via external loudspeakers. Participants were then fitted into a chin rest and the eye tracker was calibrated. Each trial began with the presentation of a visual display. Participants were allowed to freely view the display for 1,000 ms, after which the auditory stimulus began. The display remained on screen during the auditory stimulus and for an additional 1,000 ms thereafter. The participants' task was to give the correct answer by pressing a button as quickly and as accurately as possible. Answers were balanced so that *Richtig* (“True”) was the correct answer on half of the trials. Feedback was given to participants after each response by displaying (*Korrekt/Inkorrekt*, “Correct/Incorrect”). Participants initiated each new trial by button press. The experiment was implemented in Experiment Builder (SR Research, v 2.1.140) and began with three practice trials. The entire session lasted approximately 45 min.

### 2.2. Results

Analyses were conducted using the *lmer* package *lme4* library, version 1.1-10; Bates et al. (2015) in the statistics software package R (version 3.4.2; R Development Core Team, 2017). Fixed effects were contrast-coded and evaluated via likelihood ratio tests implemented in *lmerTest* (Kuznetsova et al., 2017), where denominator *df* was estimated using the Satterthwaite method. Participants and items were entered as crossed, independent, random effects. All models included maximal random effects structures (Barr et al., 2013). We report estimates, standard errors, *t* and *p*-values associated with likelihood ratio tests.

#### 2.2.1. Eye Movements

**Figure 2:**
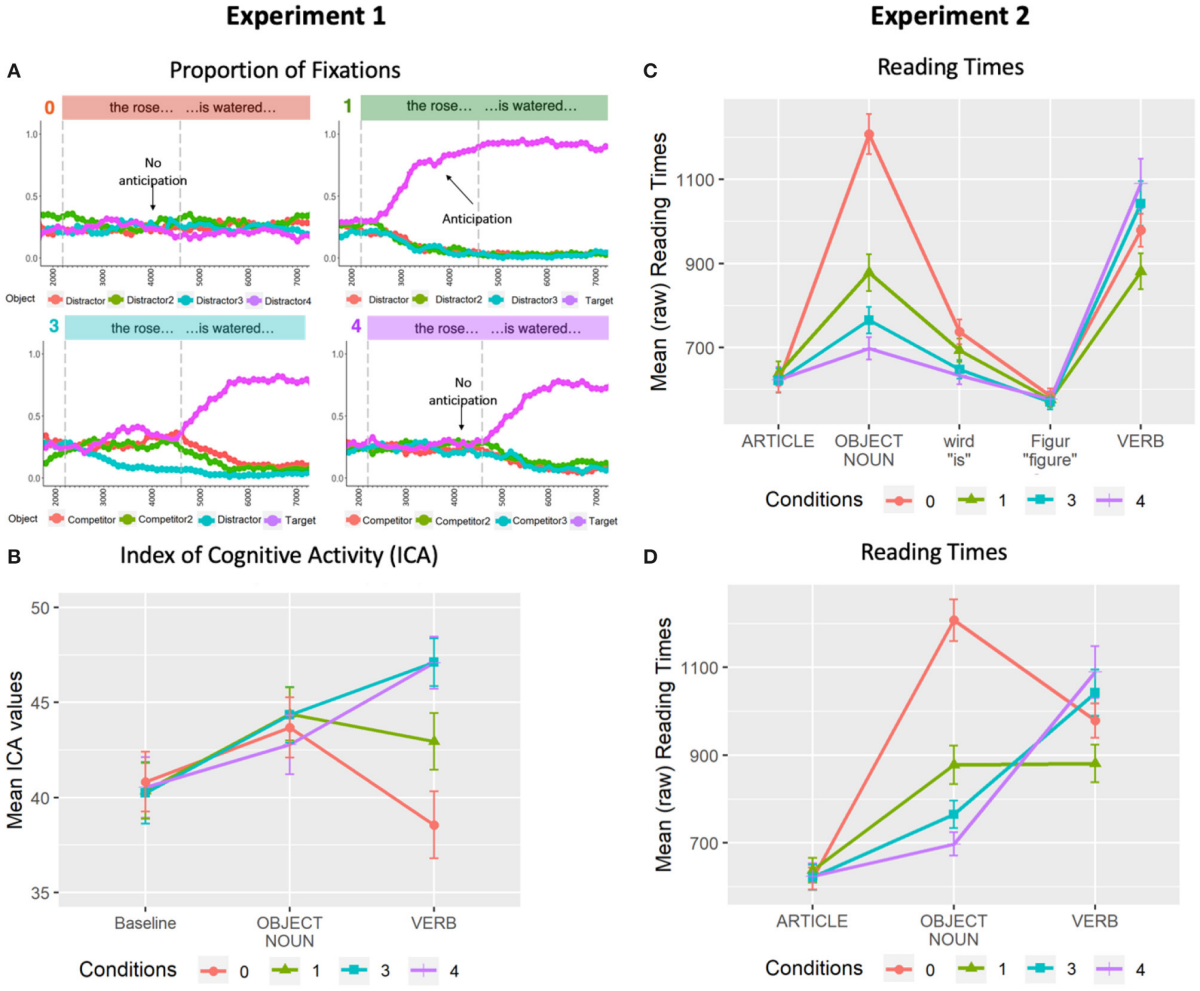
Results from Experiment 1 (left) and Experiment 2 (right). **(A)** Proportion of fixations across averaged trial length in 100 ms bins in the 0-match, 1-match, 3-match, and 4-match conditions. **(B)** Mean ICA values for all four conditions. **(C)** Mean word-by-word (raw) reading times by condition across entire sentence. **(D)** Mean word-by-word (raw) reading times by condition for critical regions only. All error bars indicate 95% confidence intervals.

For presentation purposes only, Figure 2A shows the overall proportion of fixations across an averaged trial in all conditions. Visual inspection suggests that when the visual scene allowed for the anticipation of potential target verbs (i.e., 1-match and 3-match conditions), fixations on the scenes containing the mentioned object began to increase at the onset of the noun phrase (left-most dashed vertical line). In contrast, no discrimination is possible in the noun region for the 0-match and 4-match conditions: in the 0-match condition, none of the scenes are relevant, while in the 4-match condition, all of the scenes are equally relevant.

These observations were assessed statistically by comparing whether new inspections to the target scene were detected across conditions within the *noun region* (i.e., noun phrase onset to offset: *M* = 658, *SD* = 103). The presence/absence of new inspections to each scene were encoded as a binary dependent variable and were analyzed using generalized mixed-effects regression models (GLMER) with a binomial distribution. Orthogonal comparisons between conditions were contrast coded and entered into each model as fixed effects (C1C3: 1-match vs. 3-match, C3C4: 3-match vs. 4-match) with crossed random effects for subjects and items: glmer(number_of_target_inspections ~ C1C3 + C3C4 + (1 + C1C3 + C3C4 || Subject) + (1 + C1C3 + C3C4 || Item), data, family = “binomial”). Comparisons with the 0-match condition were omitted here as there was no target scene. Results confirmed a significant increase in new inspections of the target scene during the noun region in the 1-match condition (*M* = 0.20, *SD* = 0.40) compared to the 3-match condition (*M* = 0.12, *SD* = 0.33) [β = 0.59, *SE* = 0.16, *z* = 3.77, *p* < 0.01]. In contrast, new target-scene inspections in 3-match where not significantly higher than in the 4-match (*M* = 0.09, *SD* = 0.29) condition [β = 0.32, *SE* = 0.19, *z* = 1.70, *p* = 0.09].

#### 2.2.2. Index of Cognitive Activity

ICA reflects fluctuations in the pupil signal that are due to effortful cognitive activity (Marshall, 2000). It is computed as the number of times per second that an abrupt discontinuity (i.e., an ICA event) in the pupil signal is detected, after controlling for any effects due to eye movements and the light reflex (Marshall, 2007). Low values of ICA indicate lower cognitive effort, while higher values reflect greater effort. Importantly, ICA maintains both time and frequency information and can therefore provide a fine-grained analysis of changes in cognitive effort over time.

To assess the effects of visual context on processing effort, we compared the number of ICA events across conditions for two critical regions, namely a noun and a verb region, defined as follows: Consistent with previously established methods (Ankener et al., 2018; Sekicki and Staudte, 2018), analyses for each region were conducted on non-overlapping time windows spanning 600 ms and beginning from the middle of each critical word's duration. ICA values that were 2.5 standard deviations or greater than an individual subject's mean were considered outliers and were excluded from analyses (0.02%).

Figure 2B presents the ICA results for all conditions in the critical noun and verb regions. For presentation purposes only, a baseline region (“Tell me if”) is also included. No differences can be seen in either the baseline or noun regions. However, clear differences emerge in the verb region. To assess these observations statistically, the ICA events obtained within the two critical time windows were treated as count variables and analyzed as dependent variables in separate GLMER models with Poisson distributions. The assessed contrasts were C0C1 (0-match vs. 1-match), C1C3 (1-match vs. 3-match), and C3C4 (3-match vs. 4-match). The following model was used to analyze both the noun and the verb region: glmer(ICA ~ C0C1 + C1C3 + C3C4 + (1 + C0C1 + C1C3 + C3C4 || Subject) + (1 + C0C1 + C1C3 + C3C4 || Item), data, family = poisson (link = “log”)). In the noun region, no significant differences between conditions were found (*p*s > .16). In contrast, results for the verb region revealed significantly fewer ICA events in the 0-match condition (*M* = 38.55, *SD* = 15.40) than the 1-match condition (*M* = 42.94, *SD* = 13.15) [*β* = −0.13, *SE* = 0.03, *z* = −3.76, *p* < 0.01], and significantly fewer ICA events in the 1-match condition than the 3-match condition (*M* = 47.12, *SD* = 11.22) [*β* = −0.10, *SE* = 0.03, *z* = −3.70, *p* < 0.01]. No reliable differences were found between the 3-match and 4-match (*M* = 47.08, *SD* = 12.25) conditions [*β* = 0.003, *SE* = 0.02, *z* = 0.16, *p* = 0.87].

Taken together, these results indicate that there is an effect of multimodal information on the expectedness of the disambiguating verb, and consequently on the effort required to process that verb.

### 2.3. Discussion

Results from Experiment 1 revealed that processing effort at the target verb was modulated by the number of actions in the display that were consistent with the verb (Figure 2B). More specifically, the verb was easier to process when only one verb-consistent action was displayed (1-match condition) than when three or four verb-consistent actions were shown (3- and 4-match conditions), as reflected in lower mean ICA values during the verb region. Somewhat surprisingly, the 0-match condition yielded the lowest ICA values. This finding differs from results in Ankener et al. (2018), where the equivalent condition yielded the highest values. However, in the 0-match condition in the current experiment, participants could already determine at the noun that the correct answer to the question (e.g., “Tell me if the rose.”) could only be “Yes” if the question ended with “.is missing.” Thus, listening to the verb was not required in this case, thereby making the verb in the 0-match condition the easiest to process. Finally, statistical analyses further revealed typical anticipatory eye movements during the noun region (i.e., looks only to likely upcoming actions/verbs) even though the difference in new target-inspections between the 3- and 4-match conditions did not reach significance in this study. However, as in Ankener et al. (2018), the distinct allocation of attention (1-match vs. other) did not appear to modulate processing effort at the expectation-generating word.

Taken together, these results indicate that visual context can similarly affect the predictability of both verbs and nouns. We also replicate the lack of an effect on processing effort for the word that provides the constraining information (i.e., the word that reduces referential uncertainty). Thus, regardless of word class, processing effort seems to correlate with visually-situated expectancy but not with the reduction of referential uncertainty.

## 3. Experiment 2: G-Maze Reading Times as a Measure of Noun and Verb Processing

**Figure 3:**
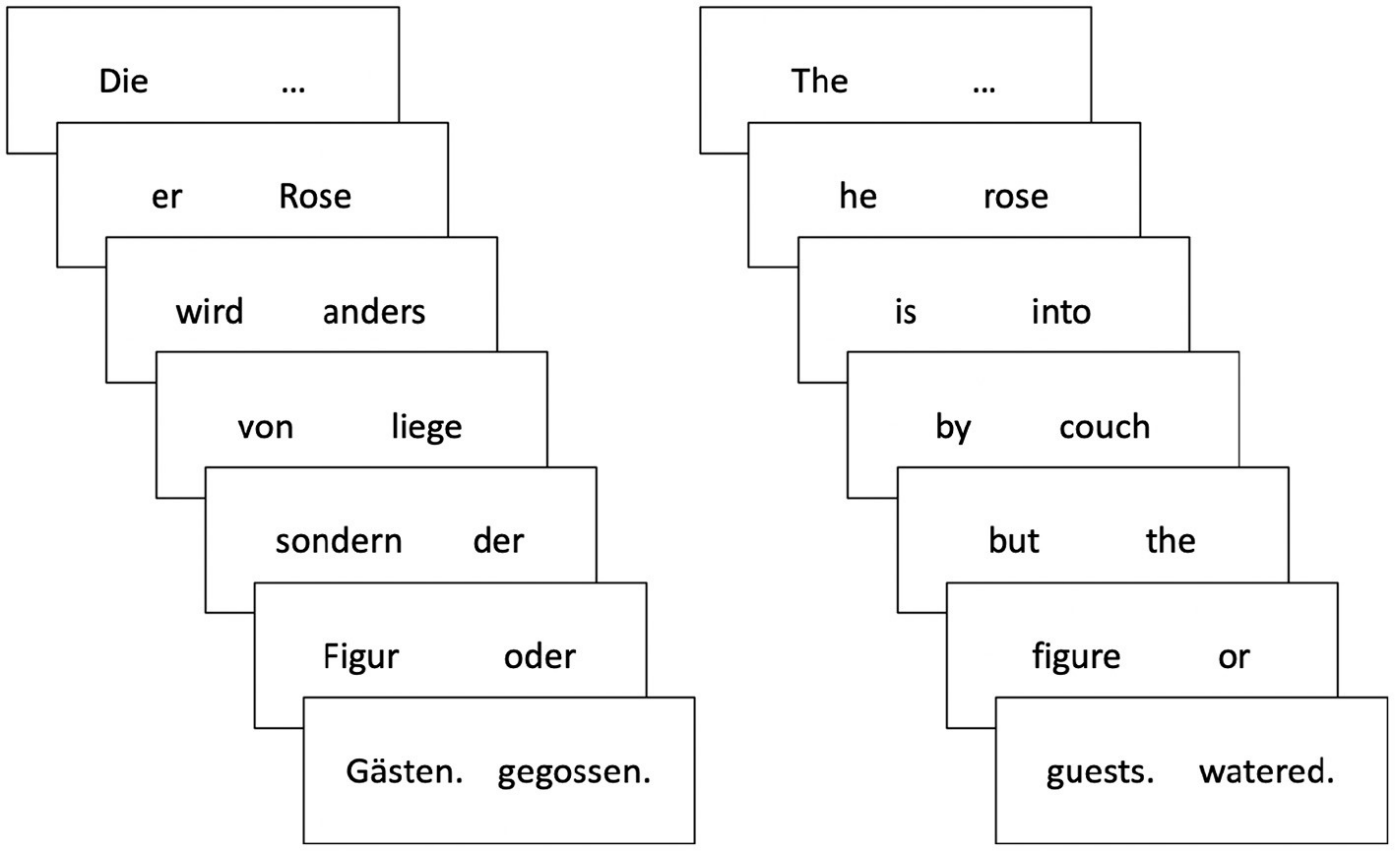
Experiment 2: Structure of the G-Maze task for the example sentence, *Die Rose wird von der Figur gegossen* **(left)**, and its rough English translation “The rose is by the figure watered” **(right)**. Sentences were presented word by word as a sequence of forced choices between two alternatives, only one of which continued the sentence grammatically.

The aim of Experiment 2 was to replicate the pattern of effects on processing effort from Experiment 1 (i.e., the influence of expectations on the processing of the reference-resolving word, and the lack of an effect on processing of the expectation-generating word) using a different dependent measure. To this end, we collected self-paced reading times using a novel combination of the visual world paradigm and the Grammaticality Maze (G-Maze) task (Forster et al., 2009). The G-Maze task is a variation of self-paced reading, which has been shown to have better precision (i.e., is less susceptible to spill-over effects) than standard forms of self-paced reading (Witzel et al., 2012), and therefore can more accurately identify the point at which processing time differences emerge during online comprehension (Sikos et al., 2017). Sentences are presented word by word as a sequence of forced choices between two alternatives, only one of which continues the sentence grammatically. If the participant successfully navigates the “maze” by choosing the correct word from each pair, the selected words form a coherent sentence (Figure 3). Specifically, we predicted that more predictable verbs would elicit less processing effort, reflected in shorter reading times. Because Experiment 1 and previous work found no impact of the reduction of referential uncertainty on processing effort, we expected to find no differences in reading time on the object noun in the current study. If, however, uncertainty reduction does modulate processing effort in the current design, then the 1-match condition could elicit longer reading times than the 3- and 4-match conditions, because it allows for greater reduction of uncertainty.

### 3.1. Methods

#### 3.1.1. Participants

Thirty-two native speakers of German (19 female; 18–28 years old, *M* = 22.3, *SD* = 2.5) who had not participated in Experiment 1 were recruited from Saarland University community and were compensated with 10€ for their participation. All participants reported normal or corrected-to-normal vision. Participants who did not successfully complete at least 70% of experimental trials, including both the G-Maze task and the subsequent truth-value judgement task, were excluded (*n* = 1). Two additional participants were excluded due to data corruption issues, resulting in a total of 29 participants.

#### 3.1.2. Materials

On each trial, participants (a) viewed a visual display consisting of four scenes, then (b) completed a G-Maze task presenting a sentence which either did or did not refer to one of the scenes in the display, and finally (c) decided whether the sentence correctly described one of the scenes or not (*Richtig/Falsch*; “True/False”). As in Experiment 1, the expectedness of the target verb was manipulated by pairing each sentence with four visual displays in a 1 x 4 design (Figure 1). The same visual displays and conditions were used as in Experiment 1. Linguistic stimuli were adapted from the materials in Experiment 1 by using an alternate template so as to be compatible with a True/False response: [artikel objekt] *wird von der Figur* [ge-verb-n] (“[article object] is by the figure [verb-ed]”). For instance, *Die Rose wird von der Figur gegossen* (“The rose is by the figure watered”). This construction also ensured that the sentence-final word (the verb) was the locus of both sentence-level integration and visual scene identification in the 1-, 3-, and 4-match conditions. Note, however, that in the 0-match condition participants could already recognize upon encountering the noun that the sentence would not refer to any of the depicted scenes. Thus, the correct response to one-quarter of the experimental items was *Falsch* (“False”).

To disguise the critical manipulation, the same 40 filler items were used as in Experiment 1, with the following modifications. First, the sentence structures were adapted so as to be compatible with the truth-value judgment task (e.g, *Zum Zeichnen benutzt die Figur den Kugelschreiber* (“The figure uses the ballpoint pen to draw”). Second, sentences varied in length from 5 to 12 words. Finally, half of the filler sentences did not correctly describe a scene in the corresponding display, either because the mentioned action or the mentioned object (as in the 0-match condition) was not present in the display, or because the mentioned object and action (which were both depicted) did not appear together in any of the scenes. The goal of these fillers was to discourage participants from basing their response only upon the presence or absence of the mentioned action and object in any of the scenes. Thus, the correct response for half of the filler items was *Falsch* (“False”). Four stimulus lists were created from the above materials using the same constraints as in Experiment 1.

#### 3.1.3. Procedure

The same general procedure was used as in Experiment 1, with the following modifications. During the familiarization session, the verb corresponding to each scene was presented visually rather than auditorily. During the experimental session each trial began with the presentation of a visual display that participants were allowed to freely view for as long as they wished. Upon pressing a button the display was replaced with the G-Maze task, which began with two crosses (+) that remained on screen for 1,000 ms, indicating where subsequent word pairs would appear. Each word in the sentence (except the first word) was then presented together with a foil word, which was not a grammatical continuation of the sentence^3^. The first word in every sentence was paired with ellipses (“…”). Presentation side (left, right) was randomized such that the correct word appeared equally often on each side. Any punctuation (i.e., comma, period) that appeared with a word also appeared with its foil. Participants were instructed to choose as quickly and as accurately as possible the word that best continued the sentence. Participants indicated their selection by pressing the left or right button on a button box, and the amount of time required for selecting the grammatical continuation was recorded as the reading time for that word. If the correct word was chosen, the next pair of words appeared automatically. However, if a foil word was selected, or if no response was given within 8 s, negative feedback (*Inkorrekt*, “Incorrect”) was displayed and the trial was aborted. Once the end of a sentence was reached, participants were asked for a truth value judgment. They used a button box to indicate whether the sentence contained a correct descriptive statement or not. For 62.5% of the trials the correct answer was *Richtig* (“True”). Feedback was given after each response (*Korrekt/Inkorrekt*, “Correct/Incorrect”). Participants initiated each new trial by button press. After half of the trials were completed, participants were given the opportunity for a short break. The experiment was also implemented in Experiment Builder and began with three practice trials. The entire session lasted approximately 60 min.

### 3.2. Results

#### 3.2.1. Accuracy

Overall performance on the G-Maze task was near ceiling. Participants successfully completed 96.0% (*SD* = 0.20) of all experimental and filler mazes. Performance on the subsequent truth-value judgment task was also high (*M* = 94.3%, *SD* = 0.23), confirming that participants were reading the sentences for meaning during the G-Maze task. Only experimental trials for which both the G-Maze task and the truth-value judgment task were completed successfully (92.2%) were included in the analyses reported below.

#### 3.2.2. Reading Times

Noun and verb reading times exceeding 2.5 standard deviations by participant were trimmed, excluding 1.9% (noun) and 2.1% (verb) of the data. The remaining noun and verb reading times were log-transformed and analyzed separately using linear mixed effects models. Orthogonal comparisons between conditions were again contrast coded and entered as fixed effects (C1C3: 1-match vs. 3-match; C3C4: 3-match vs. 4-match; C0C1: 0-match vs. 1-match). The following model was used to analyze both the noun and the verb region: lmer(log(RT) ~ C0C1 + C1C3 + C3C4 + (1 + C0C1 + C1C3 + C3C4 || Subject) + (1 + C0C1 + C1C3 + C3C4 || Item), data).

For presentation purposes only, Figure 2C presents the mean word-by-word (raw) reading times by condition. To visualize changes in processing difficulty across the entire sentence, regions are also included for the article, *wird*, and *Figure*. In order to facilitate a comparison of these results to the ICA results from Experiment 1, Figure 2D presents only the key regions. Counter to our predictions, differences between conditions first emerged at the object noun: reading times were faster for the 4-match condition than the 3-match condition (*β* = 0.10, *SE* = 0.02, *t* = 4.30, *p* < 0.001); object nouns in the 3-match condition were read more quickly than in the 1-match condition (*β* = 0.12, *SE* = 0.03, *t* = 4.12, *p* < 0.001); and object nouns in the 1-match condition were read more quickly than in the 0-match condition (*β* = 0.30, *SE* = 0.02, *t* = 13.39, *p* < 0.001). As predicted, verbs were read more quickly in the 1-match condition than the 3-match condition (*β* = −0.14, *SE* = 0.03, *t* = −5.27, *p* < 0.001). Verbs in the 3-match condition were read more quickly than the 4-match condition, although this difference did not reach significance (*β* = −0.02, *SE* = 0.02, *t* = −0.83, *p* = 0.41). In addition, verbs in the 1-match condition were read more quickly than the 0-match condition (*β* = 0.12, *SE* = 0.02, *t* = 4.79, *p* < 0.001).

### 3.3. Discussion

Figures 2B,D present the key results from both experiments side-by-side for comparison. Reading times from Experiment 2 revealed a graded effect of visual context on processing effort at the object noun. These results showed that nouns were easiest to process in the 4-match condition and became parametrically more difficult as fewer and fewer objects in the display matched the mentioned noun. Processing of the noun was most difficult in the 0-match condition. This pattern may indicate that scenes primed/preactivated the mentioned nouns in Experiment 2: Participants were asked to carefully view and remember the scenes, which were then removed before the G-Maze task began. Thus, the noun (e.g., rose) may have been more prominent—and thus potentially remembered better—when it was depicted in multiple scenes.

Reading times in the verb region largely replicated the ICA results from Experiment 1. That is, verbs were read most quickly when the noun reduced all uncertainty about which scene was being referred to, and consequently made the upcoming verb highly predictable (1-match condition). In contrast, when referential uncertainty remained, participants took longer to process the verb. However, the reading time difference between 3-match and 4-match conditions did not reach significance. This result also replicates previous findings (Ankener et al., 2018; Staudte et al., 2021) and suggests that discrimination between three and four potential target objects/scenes has relatively little impact on processing effort. Finally, when no expectations for the verb are generated because the mentioned object is not depicted in any of the scenes (0-match condition), processing time increases relative to the 1-match condition. Interestingly, however, reading times indicate that the verb in the 0-match condition is still easier to process than when there is some referential uncertainty (3- and 4-match conditions). We attribute this intermediate level of processing effort to a combination of two effects: a facilitation effect due to recognizing that the verb is not relevant for the answer (i.e., recognizing that the answer will be “False”) and an inhibition effect due to not being able to anticipate the verb, despite still having to fully process the verb in order to complete the G-Maze task (but see also the General Discussion).

## 4. General Discussion

The aim of the current studies was to investigate whether the generation of expectations for upcoming words in visually-situated language comprehension—and the resulting reduction of referential uncertainty—simply does not modulate processing effort, as suggested by Ankener et al. (2018) and Staudte et al. (2021), or alternatively, whether the lack of effects found at the expectation-generating word in these previous studies can be better explained by word category differences in the referential function of nouns and verbs in a reversed word order.

To address these questions, we conducted two visually-situated comprehension experiments, which essentially reverse the functional roles of nouns and verbs. Experiment 1 employed the visual world paradigm and ICA as a measure of processing effort. Experiment 2 sought to validate those findings using a more exploratory method in which processing difficulty was assessed via reading times in the G-Maze task.

### 4.1. Reference Resolution

In the current design, comprehenders were only able to uniquely resolve the referent upon encountering the verb. Crucially, both experiments and dependent measures revealed a similarly graded influence of situated context on processing effort at the verb: results of both studies showed an increase in processing effort as the number of depicted actions matching the verb increased. These findings largely replicate the effect found at sentence-final nouns in Ankener et al. (2018), where the noun served the role of uniquely identifying the referent. The results therefore indicate that verbs as well as nouns can be used to resolve referents in a visual scene—despite the inherent functional differences due to word category—and thereby allow the reader to recover the intended proposition of the sentence. In addition, processing of the reference-resolving word (whether it be a noun or a verb) reliably benefits when prior linguistic and visual information combine to generate concrete expectations for that word.

One obvious difference in results across Experiments 1, 2, and Ankener et al. (2018) is the pattern of effects found at the reference-resolving word in the 0-match condition. Whereas Ankener et al. (2018) found that the 0-match condition elicited the highest processing effort in this region, ICA results from Experiment 1 revealed that this condition elicited the lowest processing effort. Moreover, reading time results from Experiment 2 indicate that processing effort for the reference-resolving word in the 0-match condition was intermediate between the 1-match and 3-match conditions. However, these differences can be readily explained as a consequence of the different tasks used in each study. In contrast to Ankener et al. (2018), participants in Experiment 1 did not need to fully process the reference-resolving word in the 0-match condition in order to successfully respond to the query (e.g., Tell me if the rose that is by the figure watered is on the top/bottom/left/right/is missing). This is because the mentioned object did not appear in any of the scenes, thus it became immediately clear upon encountering the noun that the correct response could only be “…is missing.” In Experiment 2, however, while processing of the verb was not strictly necessary to successfully complete the subsequent truth-value judgment task (e.g., The rose is by the figure watered; True/False), the G-Maze task forces each word in the sentence to be accessed and integrated into the unfolding utterance representation—only then can the comprehender select the correct word instead of a foil and successfully navigate the maze to the end of the sentence. In the 0-match condition, it might be obvious that the correct response will eventually be “False,” however the verb must still be fully processed and selected beforehand. Moreover, in contrast to the 1-match condition, in which the verb can be anticipated, comprehenders in the 0-match condition do not have the benefit of visual preactivation of the verb. Thus, the combination of these two processes (i.e., facilitation in the truth judgment task and lack of preactivation) may explain why reading times for the 0-match condition in Experiment 2 fall between the 1-match and 3-match conditions.

### 4.2. Generation of Expectations

In both experiments of the current study, expectations for upcoming verbs were generated at the object noun. Consistent with Ankener et al. (2018), ICA results from Experiment 1 revealed no modulation of processing effort at the expectation-generating word. In contrast, however, reading time results from the same expectation-generating noun in Experiment 2 showed a reliable modulation of effort: processing difficulty was greatest in the 0-match condition (when the mentioned object did not appear in the display) and parametrically decreased as more potential target objects were depicted in the visual context. In fact, this pattern of effects at the expectation-generating noun appears to be the inverse of the subsequent pattern found at the reference-resolving verb, where processing difficulty was greatest in the 4-match condition and decreased as fewer potential target objects were depicted. Although this combined pattern of effects in Experiment 2 is consistent with the notion of uncertainty reduction, it is also consistent with the results of Maess et al. (2016), which argues for a direct “trade-off” in processing effort between preactivation and a later point in time when that preactivated word is encountered. On this account, the effort expended at the noun reflects preactivation of semantic features of the expected, upcoming scene description, which is then offset by a complementary facilitation in processing the subsequent (expected) verb (Maess et al., 2016). Similarly, these results are in line with the findings on pre-updating (during verb processing) and the processing trade-off with the predicted word (noun) in Ness and Meltzer-Asscher (2018).

Yet another explanation for the parametric effects found at the expectation-generating noun in Experiment 2 is that the difference in reading times may have been driven by task-based effects rather than uncertainty reduction *per se*. In adapting the materials of Experiment 1 to Experiment 2, we chose to remove the visual display during the G-Maze portion of the task. This decision was driven by two related concerns: (1) that a co-present visual scene could potentially draw the participant's gaze away from the G-Maze task, and (2) that this effect would vary systematically by condition. Accordingly, participants were instructed to view each display carefully so that they could later respond as to whether or not the sentence corresponded to one of the displayed scenes. One unintended consequence of this decision is that participants may have utilized a non-trivial amount of working memory to accomplish this goal, which may then have influenced the processing of the object noun. For instance, it is possible that some proportion of participants consciously or unconsciously labeled the objects or actions depicted in each display in order to better remember the key information. Exit survey results provide some support for this account. When asked whether they used any particular strategies in order to successfully perform the task, 15 participants reported that they tried to memorize either the depicted objects, actions, or both. If this was indeed the case, explicitly labeling objects during the preview phase of the task would presumably preactivate^4^ the mentioned noun such that its subsequent processing would be facilitated. This could therefore explain the reading time advantage for the noun in those conditions in which the mentioned object was present in the visual context (1-, 3-, and 4-match conditions) relative to when it was absent (0-match condition). Moreover, preactivation of the noun is likely to be greater when more of the scenes in the display contain the mentioned object. Thus, this account is also consistent with the parametric effects found at the expectation-generating noun.

If this final explanation is correct—and the observed noun effects in Experiment 2 are therefore specific to the procedure used in our G-maze design, wherein the visual context was removed before sentence processing began—then one of our original research questions would remain unresolved: Why were no effects of uncertainty reduction or preactivation/pre-updating observed during the processing of the constraining word, neither on the noun in Experiment 1 (noun), nor on the verb in Ankener et al. (2018) and Staudte et al. (2021)? Here we speculate that the co-present visual scene used in these latter experiments may have played a role in why processing effort was not affected in such cases, and we offer several explanations as to why this might be the case. Firstly, participants in those experiments did not necessarily need to maintain (one or more) predictions in working memory. Instead, they could simply rely on the external representations (Spivey et al., 2004) visible in the co-present visual display to mentally flag objects with regard to match vs. no-match, rather then computing and maintaining representations of all matches. Thus, processing effort might not have been affected by whether or not one or more objects/actions in the visual display served as potential verb (arguments). Secondly, the amount of referential uncertainty that is reduced when going from four to one potential objects/actions is relatively small, at least when compared to the difference between high and low constraining words in purely linguistic contexts with no co-present visual scene, as in Maess et al. (2016); Ness and Meltzer-Asscher (2018). In such cases, low constraining words allow for dozens or even hundreds of continuations (comparable to the 0-match conditions in the current studies), while high constraining words typically license only a few concrete predictions. Thirdly, the reduction of uncertainty and the maintenance of multiple predictions could each elicit processing effort, which could then cancel each other out. That is, while reducing uncertainty from four to one option might require increased effort, less effort would then be required to maintain that single object/action representation in working memory than four representations. In contrast, the comparison of processing effort across conditions in Maess et al. (2016) was not among different numbers of preactivated representations, but was instead between preactivation and the lack thereof.

All of these alternative explanations are grounded in the specifics of simultaneously perceiving linguistic and visual information. This makes Experiment 2 particularly interesting—although exploratory—because no objects were co-present and instead had to be mentally-represented, predicted, and maintained in working memory. However, further research is needed to tease apart whether the effects during noun processing in Experiment 2 do indeed index any of the above mentioned “forward-looking” mechanisms to predict upcoming content, or whether they are instead a result of preactivation based on the previously shown scenes.

## 5. Conclusion

In sum, the results of the current study indicate that verbs as well as nouns can be used to resolve referents in a visual scene, and thus to reconstruct the speaker's intended proposition. Moreover, processing of the reference-resolving word—whether it be a noun or a verb—reliably benefits from the prior linguistic and visual information that leads to the generation of concrete expectations for that word.

## Data Availability Statement

The datasets presented in this study can be found in online repositories: https://doi.org/10.7910/DVN/ST41P8.

## Ethics Statement

The studies involving human participants were reviewed and approved by Deutsche Gesellschaft für Sprachwissenshaft (DGfS). Participants provided their written informed consent prior to participation.

## Author Contributions

LS, KS, and MS conceived and designed the studies and analyses and contributed to the manuscript. KS collected the data. All authors contributed to the article and approved the submitted version.

## Conflict of Interest

The authors declare that the research was conducted in the absence of any commercial or financial relationships that could be construed as a potential conflict of interest.

## Footnotes

^1^The 1-, 3-, 4-, and 0-match conditions were chosen to facilitate a comparison of the current results with Ankener et al. (2018), Experiment 4. The authors of that study chose the 1- and 4-match conditions in order to maximize the difference in expectation across conditions while simultaneously keeping the overall visual complexity of the displays low. The 3-match condition was chosen in order to allow visual displays to be counterbalanced across items.

^2^Because the experimental items did not contain a 2-match condition, we included more filler items in which two scenes contained the mentioned object in order to approach an equal balance of 1-, 2-, 3-, 4-, and 0-match items across the entire study.

^3^Foils were created in a two-stage process following Sikos et al. (2017). First, a custom Python script randomly selected a foil candidate for each word in each experimental and filler item. Foil candidates were constrained such that they did not appear in bigrams with the correct word at the previous position in the sentence within a large German corpus. Second, each foil was then hand checked by a trained native-German linguist to ensure that it was not a grammatical continuation of the sentence. The same foil was used for identical words across conditions.

^4^Note that preactivation here refers to the noun being preactivated through viewing and memorizing the preceding visual display as opposed to the noun preactivating the upcoming verb (al features).
